# Error Analysis of Deep Sequencing of Phage Libraries: Peptides Censored in Sequencing

**DOI:** 10.1155/2013/491612

**Published:** 2013-12-12

**Authors:** Wadim L. Matochko, Ratmir Derda

**Affiliations:** Department of Chemistry and Alberta Glycomics Centre, University of Alberta, Edmonton, AB, Canada T6G 2G2

## Abstract

Next-generation sequencing techniques empower selection of ligands from phage-display libraries because they can detect low abundant clones and quantify changes in the copy numbers of clones without excessive selection rounds. Identification of errors in deep sequencing data is the most critical step in this process because these techniques have error rates >1%. Mechanisms that yield errors in Illumina and other techniques have been proposed, but no reports to date describe error analysis in phage libraries. Our paper focuses on error analysis of 7-mer peptide libraries sequenced by Illumina method. Low theoretical complexity of this phage library, as compared to complexity of long genetic reads and genomes, allowed us to describe this library using convenient linear vector and operator framework. We describe a phage library as N × 1 frequency vector *n* = ||n_*i*_||, where n_*i*_ is the copy number of the *i*th sequence and N is the theoretical diversity, that is, the total number of all possible sequences. Any manipulation to the library is an operator acting on *n*. Selection, amplification, or sequencing could be described as a product of a N × N matrix and a stochastic sampling operator (**S** **a**). The latter is a random diagonal matrix that describes sampling of a library. In this paper, we focus on the properties of **S** **a** and use them to define the sequencing operator (**S** **e** **q**). Sequencing without any bias and errors is **S** **e** **q** = **S** **a** I_N_, where I_N_ is a N × N unity matrix. Any bias in sequencing changes I_N_ to a nonunity matrix. We identified a diagonal censorship matrix (**C** **E** **N**), which describes elimination or statistically significant downsampling, of specific reads during the sequencing process.

## 1. Introduction

*In vitro* selection experiments—such as phage display [1, 2], RNA display, SELEX, and DNA aptamer selection [3, 4]—employ large libraries, from which 10^2^–10^6^ active sequences are identified through iterative rounds of selection and amplification. With the recent emergence of deep sequencing, it became possible to extract a large amount of information from the libraries before and after selection [5–10]. Deep examination of the library is a promising technique for direct evaluation of binding capacities of all binding sequences from one panning experiment. Deep sequencing also allows the characterization of unwanted phenomena in selection, such as amplification bias [6, 11].

Analysis of 10^6^ reads by deep sequencing gave rise to a large number of errors that were not present in the analysis based on the small number of sequences obtained using the Sanger method. Analysis of errors in information-rich datasets is a problem with over 50 years of history; correction of digital data made of bits or words is a topic of intense research in communication theory [12]. As phage display operates with limited digital sets, data analysis techniques from the communication theory could be applied to phage display. For example, Rodi and coworkers used a positional frequency matrix to calculate the informational content or Shannon entropy of each sequence [13]. This approach could be used to distinguish potential fast growing sequences from potential hits [14]. With the introduction of deep sequencing, the problem of error analysis in phage display becomes identical to a classical information theory problem: “reproducing at one point, either exactly or approximately, a message selected at another point” [15]. The “message” is the sequence information stored in the library. Sequencing process transmits this information and makes either stochastic or predictable errors. Understanding the sources of errors during sequencing could provide mechanisms for bypassing them, for correcting the errors, and for maximizing the amount of useful information received from sequencing.

There are over 10,000 published literature reports that contain the terms “deep sequencing” or “next generation sequencing” or any of the trademark names such as “Illumina” (reference: ISI database). Among these reports, less than 10 published reports describe sequencing of phage-displayed libraries [5–7, 9, 10, 16–19]. Deep sequencing efforts in the literature are largely focused on genome assembly and metagenomic analyses. The error analysis techniques tailored for genome assembly cannot be used directly for analysis of phage libraries because the data output from phage library sequencing is very different from the genome assembly. In genome assembly, genomic DNA is shredded into random fragments and sequenced. The genome is then assembled from these fragments *in silico*. Although multiple fragments cover each area of the genome, the probability to observe two identically shredded fragments is very small. Two exact sequences, thus, could be considered amplification artifacts and removed by error analysis software. On the contrary, in phage-display sequencing, the reads are exactly of the same length. Duplication of the same read is important for validation of the accuracy of this read. Some researchers focus exclusively on reads that have been observed multiple times and discard singleton reads as erroneous [5]. Within each library, the copy numbers of sequences range continuously by six or more orders of magnitude [5, 6, 9]. Some phage clones are observed in the entire library only a few times; other clones could be present at copy number of 100,000 per sequencing run [5, 6, 9]. Unlike multiple cells with identical genomes, each screen is unique: identical set of sequences with identical copy numbers cannot be obtained even if the screen is repeated due to stochastic number of the screen that contains low copy number of binding clones [20].

Metagenomic analyses of microorganisms recovered from environmental samples [21, 22], also known as “microbiome” [23] and “viriome” analyses [24], encountered similar problems to those observed in phage library analysis: the concentration of species observed in a particular sample is unequal [25]. The abundance of species might range by a few orders of magnitude [26]. It is possible that error analysis tools developed in the above areas could find use in phage display sequencing. For example, there are multiple published algorithms for removing errors from low copy number reads to ascertain that low copy number sequences are new species and not sequencing errors (e.g., see [27–29] and references within). Metagenomic analysis is usually more complex than analysis of phage-display libraries. First, in metagenomics, the bacterial or viral genes must be assembled from short reads *de novo*. Second, there is no simple relationship between phylogenetic classification of “species” and the observed DNA sequence. Third, the exact number of species in the environment is unknown. On the other hand, sequencing of phage-displayed peptide libraries has none of these problems: (i) it requires no assembly steps because each sequence is covered by one read; (ii) a unique DNA sequence defines a unique “species”; and (iii) the theoretical complexity in synthetic libraries is known exactly. For small libraries, such as the library of 7-mer peptides, the complexity, (20)^7^, is within the reach of next-generation sequencing. We see phage-displayed peptide libraries as an ideal model playground for the development of optimal error analysis and error correction protocols. It is possible that error analysis developed from phage libraries analysis could then be used in other areas such as genomic and metagenomic analyses.

The errors in sequencing could be divided into “annotated” and “invisible.” The “annotated” errors that originate from misincorporation of nucleotides are annotated using Phred quality score [30]. These annotated errors are removed during the processing (see below). Examples of “invisible” errors are sequence-specific frame shifts that lead to emergence of truncated reads during the Illumina sequencing [31]. Invisible errors could also originate during the preparation of the libraries for sequencing. Examples are removal of AT-rich fragments during purification of dsDNA [32] and erroneous incorporation of nucleotides during PCR [33, 34]. Mutations have the most significant impact on the observed diversity of the library. There are 63 ways to misspell a 21-mer-nucleotide sequence with a one-letter error (point mutation). The large dynamic range in concentrations of clones in the phage library exacerbates the problem. Clones that are present in high abundance—10^5^ copies per read—are more prone to yield errors [6]. For example, we observed that random point mutations convert several short sequence with a copy number of 10^5^ to a library of sequences with copy numbers ranging from 1 to 10^2^ [11]. In attempt to unify error analysis into one convenient theoretical framework, we generalized all errors as follows. All errors either lead to disappearance of particular sequence or its conversion to another sequence of the same length. Errors, thus, operate within a finite sequence space, and it should be possible to use elementary linear algebra to generalize most processes that lead to errors.

## 2. Theoretical Description

See Table 1.

### 2.1. Operator Description of the Phage-Display Library and Selection Process

**Figure 1:**
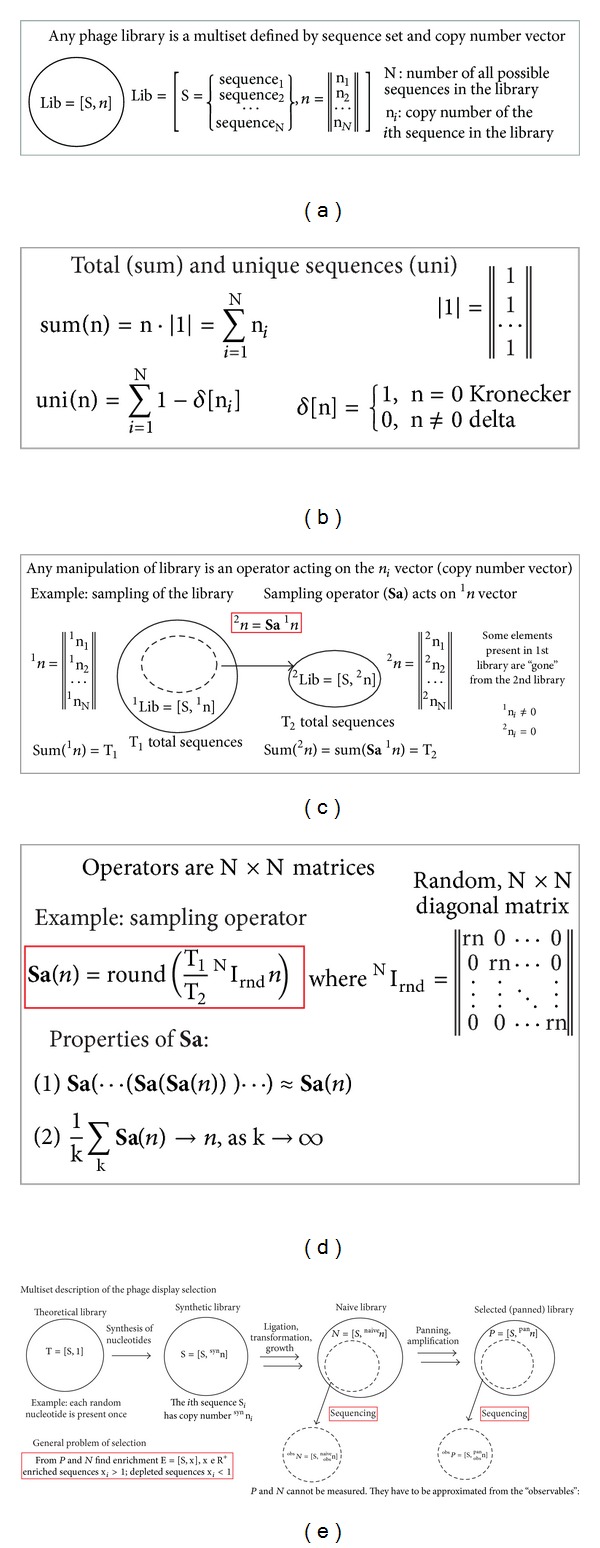
(a) Phage library can be described by multisets made of S = {sequence  set} and *n* = ||vector  of  copy  numbers||. Any change to the library can be described as function/operator acting on the *n*. (b) Relevant functions are calculations of total sequences (sum) and unique sequences (uni). (c) Any transformation of library to another library is an operator acting on *n*. Sampling of libraries to yield a sublibrary is the most important operator. (d) It can be described as N × N matrix. Specifically, **S** **a** is a diagonal matrix of values derived from random distribution. Rounding function is necessary to ensure the physical meaning of the sampling results. **S** **a** acting on the same vector yields one of many vectors that have the same number of total elements. As a consequence, **S** **a** is nonlinear, nondistributive, and noncommutative operator. Average of many **S** **a** operators is a scalar (dilution factor). (e) Any screen of any library can be described as operators acting on the copy number vectors of the naïve (or theoretical) library. Copy number vectors cannot be observed directly. They have to be measured through sequencing. As sequencing contains sampling process (**S** **a** operator), the result of sequencing is nondeterministic. Sequencing yields one of many possible *observed* copy number vectors, none of which are equal to the *real* copy number vector.

In our previous reports, we described the phage library as a multiset, or a set in which members can appear more than once [35]. This description also simplifies the analysis of the errors in these libraries. The multiset description represents a library with N theoretical members as an ordered set of N sequences and N × 1 copy number vector (*n*) with positive integer copy numbers (Figure 1(a)). Any manipulation of a phage library—such as erroneous reading or selection—changes the numbers within the copy number vector. All manipulations to the multiset, thus, could be described by operators (**O** **p**) that convert vector *n*_1_ to another vector *n*_2_ as *n*_2_ = **O** **p** *n*_1_ (Figure 1(c)). For an N × 1 vector, the operator is N × N matrix. If elements are selected or eliminated independently of one another, the N × N matrix is diagonal (Figure 1(d)). This approach is uniquely convenient for libraries of short reads. For example, a library of 7-mers contains exactly 20^7^ = 1.28 × 10^9^ peptides and is described completely using a 10^9^-element vector. This size is accessible to the computational capacity of most desktop computers.

In operator notation, phage display can be described as

(1) $$\begin{array}{c} \text{Sel}=Pan\;\;\text{Naive}, \end{array}$$

where *Naive* is the copy number vector for naïve library, *Sel* is the copy number vector after panning, and **P** **a** **n** is a panning operator. In standard phage display, the **P** **a** **n** operator is a complex product of all manipulation steps (binding, amplification, dilutions, etc.). If a screen uses no amplification and uses deep-sequencing [9, 16], or large-scale Sanger sequencing [36, 37] to analyze the enrichment, it might be possible to define the panning process as a simple product of two operators as follows:

(2) Pan=Saf Ka,

(3) Sel=Saf Ka Naive,

where **K**_**a**_ is a deterministic “association” operator, which contains association constants for every phage clone present in the library. Description of such operator is beyond the scope of this paper and we recommend consulting other reports that attempted to generalize the selection procedure [20]. Another operator in (3) is a sampling operator (^f^ **S** **a**), which describes stochastic sampling of the library with m sequences to yield a sublibrary with f*m-sequences, where f ∈ [0 1] is a sampling fraction. ^f^ **S** **a** operator has the following properties, which emanate from physical properties of the sampling procedure:

(I) (4) Saif0=0(sampling does not create new   sequences from nonexisting sequences).
(II) ^f^ **S** **a** is a diagonal operator with diagonal scalar functions ||Sa_11_  Sa_22_ ⋯ Sa_NN_||, Sa_i_(0) = 0.
(III) In *B* = ^f^ **S** **a** *A*, *B* is a vector of positive integers, B_*i*_ ≥ 0 and sum(*B*) = f*sum(*A*). Integer values ensure that the observable values of the operator have physical meaning. The clone could be observed once (1), multiple times (2, 3, etc.), or not observed at all (0).
(IV) **S** **a** is nondeterministic operator. When applied to the same vector, the operator does not yield the same result but one of the possible vectors that satisfy rules (I–III). The majority of the solutions of the operator, however, reside within a deterministic confidence interval ^f^ **S** **a** *A* ∈ [^lo^ *C* ^hi^C].
(V) As a consequence from (IV), operator **S** **a** is nonlinear, noncommutative, and nondistributive.
(VI) Large sum of sampling operators with same f should “average out” to yield I_N_ unity matrix
  (5) (Sa1f+Sa2f+Sa3f+⋯Sakf)k→f∗IN,               as  k→∞.

The **S** **a** operator is simple to implement as a random array indexing function in any programming language (e.g., see Supplementary Schemes S1, and S2 available online at http://dx.doi.org/10.1155/2013/491612). It might be possible to express ^f^ **S** **a** analytically for any f as a diagonal matrix (Figure 1(d)). In this paper, we use numerical treatment by an array sampling function because it is more convenient for multisets of general structure. We tested the random indexing implementation to show that the sampling algorithm yields a normal distribution for a large number of samples (Supplementary Figure S1). Despite the simplicity of ^f^ **S** **a** implementation—the entire code is <30 lines in MatLab—the script allows rapid calculation of the results of ^f^ **S** **a** for a multiset of reasonable size (several million sequences, Figures 4 and 5) on a desktop computer.

We evaluated the behaviors of ^0.5^ **S** **a** for several multisets. The probability to observe a specific solution is described in Figure 3(b). Individual solutions can be represented as lines with nodes on *XY*-plane, where each node represents one element of the multiset (Figures 3(d) and 3(e)). The most probable solutions reside near the “expected solution” (represented as dotted line), and the probability to observe a solution where many elements deviate from the probable solution is low (Figure 3(e)). Graphical representation of the solutions highlights that sampling could lead to deviation of the frequency of the individual elements of the multiset; for example, Figure 3(e) describes >2 fold deviation from the expected value for one of the elements. Figure 3(f) shows that the solution in which two elements deviate by >2 fold is improbable. This observation is a simple consequence of the multiplicity of the probabilities (large deviation from the average has probability *p* and the probability to observe this deviation twice is *p* ^2^).

Even in small multisets, such as {A(1)  B(2)  C(3)  D(4)} made of four unique and 10 total elements, ^0.5^ **S** **a**{A(1)  B(2)  C(3)  D(4)} operation yields large number of solutions with equal probability, termed as redundant solutions (e.g., solutions that have equal probability in Figure 3(b)). Redundancy depends on the structure of the multiset (Figure S2). This redundancy makes the calculations of all probable solutions of **S** **a** impractical. For sets even with 5-6 unique elements, identification of all vectors *B*, which satisfy equation *B* = ^f^ **S** **a** *A* and reside within a 95% interval, requires hundreds of thousands of iterations (Figure S2 and S3). On the other hand, calculation of the confidence interval of each element *B*_i_ of the vector *B* converges rapidly. A multiset {A_1000_} = {A_1_(1)  A_2_(2) ⋯ A_1000_(1000)} with 1000 unique elements and 1 + 2 + 3 + ⋯+1000 = 500,500 total elements is similar to an average deep sequencing data set (Figure 4). Calculation of all probable solutions of ^0.5^ **S** **a**{A_1000_} is beyond the capabilities of most computers. However, the 99.9% confidence interval of all elements of vector *B* = ^0.5^ **S** **a**{A_1000_} can be calculated in ~2 minutes on an average desktop computer. The red dots in Figure 4 are ^lo^ *C*_*i*_ and ^hi^ *C*_*i*_ or the 99.9% high and low confidence interval of all elements B_i_ (Figure 4).

The sampling operator is critical in phage display because sampling of libraries occurs in every step of the selection and the preparation of libraries for sequencing. The stochastic nature of sampling operators makes two identical screens “similar within a confidence interval.” Solving (1) exactly is not possible, but it should be possible to estimate the solution within a confidence interval. Consider

(6) Sel∈[Kalo Naive;  Kahi Naive],

where ^lo^ **K**_**a**_ and ^hi^ **K**_**a**_ are diagonal matrices of the upper and lower confidence intervals for the association constants. A simulation of the behavior of the **S** **a** operator (Figures 3 and S3) suggests that the relative sizes of the confidence intervals might be impractically large when the copy numbers of sequences are <10.

Multiple sampling events of the **S** **a** operator yield a normal distribution for each element of the vector (Figure 3). Fitting this normal distribution could yield a “true” value of the process. This process is identical to the extrapolation of the average from the normal distribution of noisy data. Multiple algorithms for such extrapolation exist for one- and multidimensional stochastic processes [38, 39]. We believe that **S** **a** behaves as a one-dimensional stochastic process and it might be possible to extrapolate the true value of the sampling from 7 to 10 repeated instances of **S** **a** (i.e., the number of data sufficient to fit an 1D normal distribution). The necessary practical steps towards solving (3) or (10) are the following. (i) Eliminate or account for any bias not related to binding (e.g., growth bias). (ii) Repeat the screen several times. (iii) Measure all copy numbers of all sequences, including zero values, with high confidence. Requirement (i) has been an ongoing effort in our group [11, 40] and other groups [13, 41–43]; for review see [11, 44]. Deep sequencing makes it simple to satisfy requirement (ii) and obtain multiple instances of the same experiment. For example, we described the Illumina sequencing method that allows using barcoded primers to sequence 18 unrelated experiments in one deep sequencing experiment [45]. We recently scaled this effort to 50 primer sets and evaluated the performance replicas of simple selection procedures (in preparation).

The measurement of the copy numbers of sequences is a separate problem that can be described using the same sampling operators and bias operators that describe how the library is skewed by each preparation step. For example, isolation of DNA by gel purification disfavors AT-rich sequences, whereas PCR favors sequence with within specific GC-content range [32]. The *real* sequence abundance in any phage library (^real^ *n*), hence, has to be derived from the *observed* sequence abundance (^obs^ *n*) by solving this equation:

(7) nobs=(Saf5 An)(Saf4 Seq)(Saf3 PCR) ×(Saf2 Is)(Saf1 Gr)nreal.

**Figure 2:**
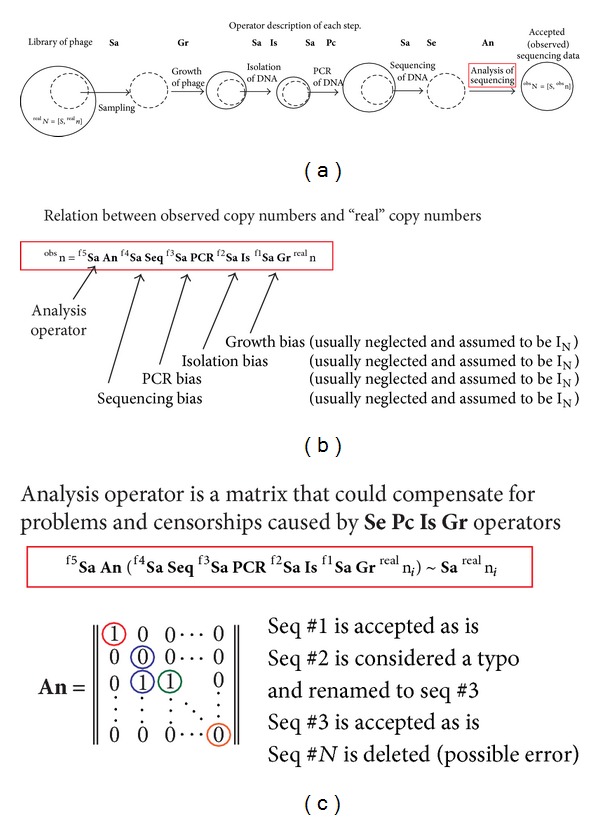
Operator description of the deep sequencing process. (a) A library of phage must be processed before deep sequencing. Each step involves sampling, which is either a deliberate partitioning of the sample or random loss of the sample. Each sample preparation state could (and does) introduce bias in sequence abundance. Each step, thus, is an operator chat changeing the *n* vector. (b) If we ignore bias during preparation, operators could be approximated as unity vectors, and sequencing could be represented as a product of sampling and analysis operators. (c) Analysis operator (**A** **n**) is a binary decision matrix, which describes what sequences are and are not considered as errors. Decisions, such as removal of sequences or correction of sequences, are the most important because they decide which “*observed*” sequences are considered “*real*.” To make the analysis of the selection process meaningful, the same **A** **n** operator should be used in all analyses.

**Figure 3:**
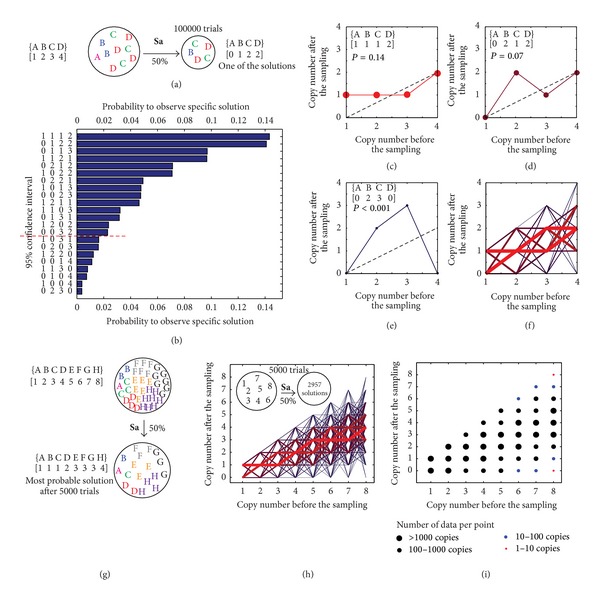
(a) Testing the sampling operator implemented as random indexing function using a model multiset. (b) In 100,000 trials, we observed 22 unique solutions from which 14 resided in a 95% confidence interval. Solutions with 0 and 1 copies of element A were found at equal abundances (“redundant solutions”). (c) Representation of the most probable solution as a line with 4 nodes; “*p*” is a probability to find the solution; dotted line is an expected “average solution” for 50% sampling. (d) The 5th most probable solution; (e) least probable solution deviates the most form the average; (f) combination of all solutions. Red thick lines describe the most probable solutions; thin blue lines describe the least probable solutions. (g) Sampling of larger multisets yields more possible solutions (here, 2957 in 5000 trials). (h) All solutions of the sampling represented as lines. (i) Probability to observe a particular copy number after sampling. While (h) is the most accurate representations of the confidence intervals, the thin blue lines describe solutions outside the confidence interval; this representation is impractical due to large number of redundant solutions in larger multisets. In (i), confidence interval could be extrapolated from distributions of individual copy numbers (e): red dots are on or outside the confidence interval.

**Figure 4:**
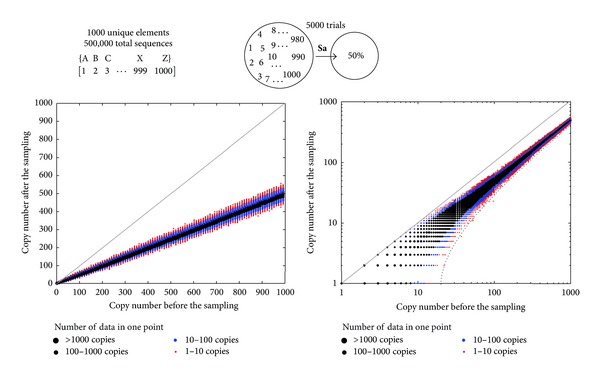
(a) Testing the sampling operator using a large multiset made of 1000 unique elements with 1000 different copy numbers. Images describe linear and log-scale representation of the confidence interval of the sampling operator. Solutions beyond this interval were not observed in 5000 trials. Dotted line represents an overestimate of the 99.9% confidence interval (for details, see Figure S4). Most probable outcomes of the **S** **a** operator have either zero or one unique sequence beyond this interval. This line is used in subsequent sections (Figures 5 and 6). We note that distributions of the copy numbers have well-defined shape; according to central limit theorem, it is a normal distribution. With enough replicas, it should be possible to extrapolate the center of this distribution, define the solutions explicitly, and bypass the stochastic nature of the **S** **a** operator.

In this equation, each operator in brackets describes a bias at a particular step. ^f^ **S** **a** describes sampling at that step, and f1–f5 describe the sampling fractions. The bias in growth (**G** **r**), isolation (**I** **s**), PCR amplification (**P** **C** **R**), and sequencing (**S** **e** **q**) could be related to the nucleotide sequences. The **A** **n** analysis operator is a matrix that describes retaining, discarding, or correcting the sequence (Figure 2(b)). An ideal **A** **n** operator could compensate for the biases introduced by another operator (Figure 2(c)). To define such operator, (7) could be potentially solved using repeated sequencing of a well-defined model library. In the next applied section, we examine the real deep sequencing data and identify conditions under which these operators could be at least partially defined.

### 2.2. Analysis or the Error Cutoff in Deep Sequencing Reads

All next-generation sequencing techniques provide quality score (Phred Score) for every sequenced nucleotide. In Illumina sequencing, this score is related to the probability of the nucleotide being correct [46]. In low throughput Sanger sequencing, the Phred score monotonously decreases with read length and the mechanisms that yield errors in capillary electrophoresis are well understood. Common practice in Sanger sequencing is to discard all reads after the first nucleotide with a Phred score of 0. In next-generation sequencing, the filtering of the reads is usually more stringent as follows.

- Discard reads that have at least one read that has score lower than “cutoff.”
- Discard reads that had cumulative Phred score lower than cutoff.
- Use a combination of A and B (accept reads with minimal cutoff and minimal cumulative score).

Many of the error analyses in the area of deep sequencing are designed for genetic reads, which have variable lengths and unknown sequence throughout the whole read. Analysis of the reads in a phage-display library is a simpler problem because phage-derived constant adapter regions flank the variable reads. Identification of the adapter region is a necessary first step in the analysis. Reads, in which the adapters cannot be mapped, cannot be used. We designed algorithms to recover reads, even if adapters were hampered by truncation, deletion, or mutation [6]. We observed that the reads flanked by the erroneous adapters had a significantly higher error rate than reads flanked by “perfect” adapters (unpublished). Example of mapping of flawed reads in Illumina sequencing is provided in ERROR_TAG_data0001.txt (see Section 4). In the remaining sections, we analyze the population of the sequences preceded by a “perfect” adapter to identify possible sequence-specific biases.

**Figure 5:**
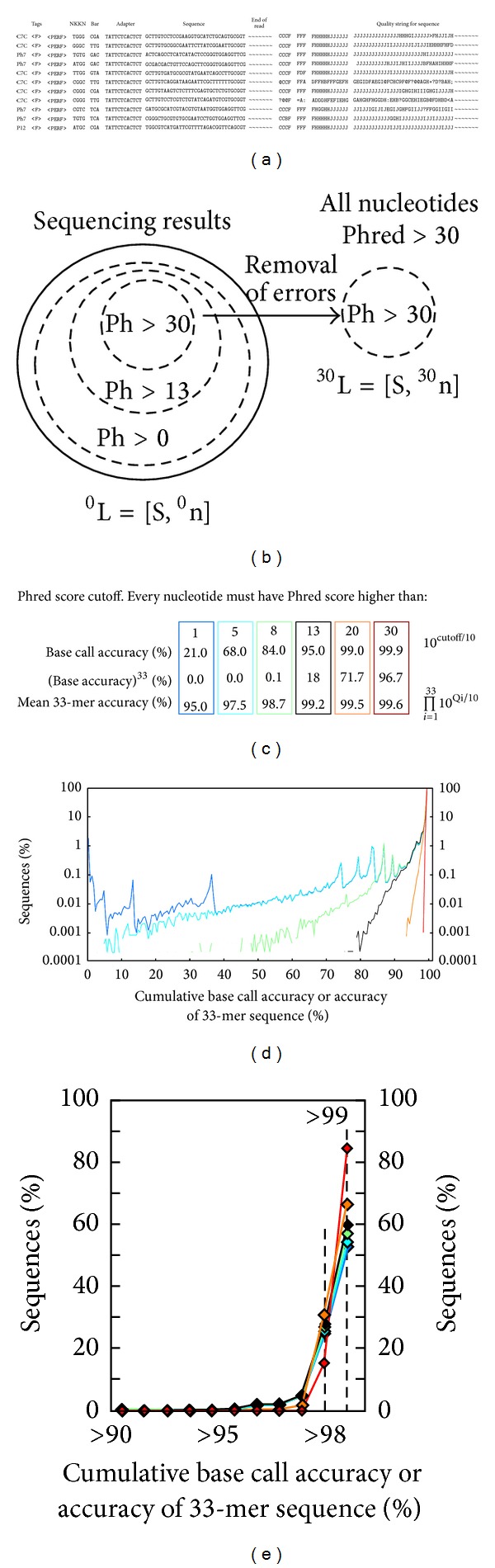
(a) Representative lines from the intermediate file from Illumina deep sequencing analysis (for more information see ERROR_TAG_data0001.txt in Section 4 and our previous publication [6]). The reads have been parsed to identify adapters and barcodes. Each read has been tagged according to the library type, direction of the read, and quality of the adapter regions. We use this intermediate library to identify reads that harbor erroneous nucleotides. (b) Multiset view of the intermediate library. The library contains subsets that have low, medium, and high quality reads. Error filtering of this intermediate library to eliminates any read with Phred score below 30 yields a high quality library of reads ^30^L. (c) Mean accuracy of the reads in the library after error filtering ranges from 95% to 99.6%. Even for very low-quality cutoff, Phred > 1, the average read quality is 95%. (d) Distribution of cumulative read accuracy in libraries processed using different cutoffs. (e) Linear plot of the data presented in (d) with zoom in on the region with >90% cumulative accuracy.

We analyzed a typical library sequenced by Illumina using various cutoffs (Figure 5). We analyzed a 33-bp segment of the library that contained variable seven amino acids and a constant region and GGGS terminus. A simple cutoff that discards reads with Phred <1 nucleotides yields library termed ^1^ *n*, which had an average 95% accuracy of the 33-nucleotide read. Reads that do not contain Phred = 0 nucleotide rarely contain multiple low-quality reads. The ^1^ *n* library was bimodal: 80% of the reads had overall accuracy of 99%, very few reads with accuracy 5–90%, and significant number of reads with accuracy of 1% (Figures 5(d) and 5(e)). These observations suggest that reads can be divided into (i) reads free of errors and (ii) reads with multiple errors.

An example of a more stringent cutoff is elimination of reads with Phred <13 nucleotides; this process yielded a library ^13^ *n* in which every nucleotide had >95% confidence. The number of total reads in ^13^ *n* was 10% less than number of reads in ^1^ *n*; that is, sum(^13^ *n*) = 0.9sum(^1^ *n*). The observed average read accuracy of the read in the ^13^ *n* library was 99.2%. Theoretically, the 0.95 confidence cutoff in a 33-mer nucleotide could yield reads with accuracy as low as (0.95)^33^ = 18%. In practice, the probability to find reads with multiple nucleotides of 95% accuracy was vanishingly small. Specifically, among 500,000 reads, the lowest observed cumulative accuracy was 77%. Such a result, for example, could be obtained in a sequence that has 27 “perfect” nucleotides and 5 nucleotides with a Phred = 13 score: (1)^27^(0.95)^5^ = 0.77. Applying the most stringent cutoff to eliminate all reads with a Phred < 30 yielded a library ^30^ *n* in which every nucleotide had 99.9% confidence. The average confidence of the reads improved subtly from 99.2% to 99.6%. The number of total reads in ^30^ *n* was 30% less than number of reads in ^13^ *n*; that is, sum (^30^ *n*) = 0.7sum(^13^ *n*). It was not clear whether such cutoff is an improvement or a detriment for analysis. In the next section, we examined how frequency of the members of the library changed upon application of each error cutoff.

### 2.3. Example of Error Analysis: Sequence-Specific Censorship during Phred Quality Cutoff

If errors occur by random chance, they should be uniformly distributed in all sequences. Removal of erroneous read, in that case, should be identical to sampling of the library by ^f^ **S** **a** operator, where f is the sampling fraction. For example, consider the removal of Phred < 13 nucleotides from an unfiltered library (process denoted as ^1^ *n* → ^13^ *n*). From the experiments, we know that sum(^13^ *n*) = 0.9sum(^1^ *n*); if errors were distributed in sequences at random, the ^1^ *n* and ^13^ *n* vectors should be related as

(8) n13=Sa0.9(n1).

The solutions should reside within a confidence interval

(9) n13∈[CloChi].

If errors occur preferentially in specific reads, the frequency of these reads should occur beyond the confidence interval of the ^0.9^ **S** **a**. This process could be described by a diagonal matrix **B** **i** **a** **s** as

(10) n13=Sa0.9(Bias(n1)).

The elements of the diagonal matrix **B** **i** **a** **s** = ||B_*ii*_|| could be estimated as follows:

(11) ni13∈[CiloCihi],  Bii=1,

(12) ni13<Cilo,  Bii=ni13(0.9ni1).

**Figure 6:**
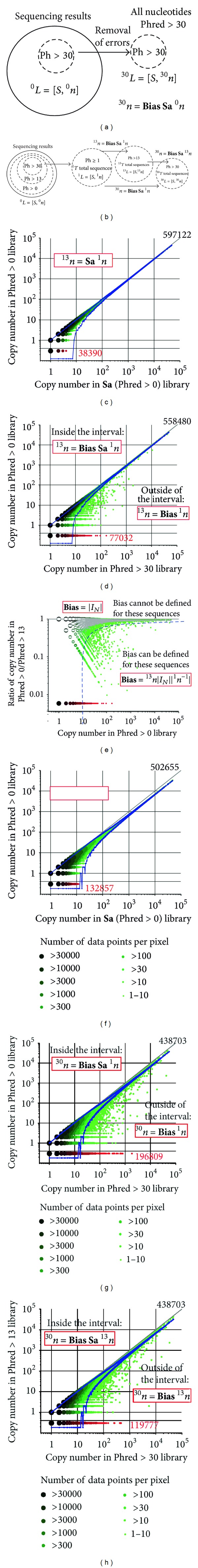
(a) Operator and multiset description of the error filtering procedure. Applying a Phred > 30 cutoff to library filtered by Phred>1 cutoff (^1^n) yields a subpopulation of the library (^30^n). If errors are sequence-independent, the ^1^n → ^30^n process should be identical to random sampling (^30^n = **S** **a** ^1^n). Any sequence-specific bias (**B** **i** **a** **s**) should be detected as deviation from **S** **a** ^1^n. (b) Progressive sampling with more stringent cutoff. (c) Theoretical **S** **a** ^1^n and theoretical 99.9% confidence interval (blue). (d) Observation of statistically significant deviation from **S** **a** operator: dots beyond the blue line represent sequences prone to bias. Red dots represent sequences that disappeared after in ^1^n → ^30^n process or during **S** **a** ^1^n sampling. (e) Magnitude of the bias range from 5 to 100-fold. (f) Bias in sampling of Phred > 30 data from Phred > 1 data ((f) is theory, (g) is observed). (h) Bias upon sampling of Phred > 30 data from Phred > 13. Many sequences were lost in this sampling and this loss was statistically significant beyond the 99.9% interval. This result shows that some sequences have propensity to harbor low- and medium-quality reads. Distribution of the errors is sequence specific.

Figure 6(c) describes the representative solution of the ^0.9^ **S** **a**(^1^ *n*) (green dots) and the confidence interval (blue lines). Supplementary Scheme S3 describes the script that calculated this interval from multiset ^1^ *n*, described as a plain text file PhD7-Amp-0F.txt, using 10,000 iterative calculations of ^0.9^ **S** **a**(^1^ *n*). This calculation required ~2 hours on a desktop computer. Confidence interval was estimated as the minimum and maximum copy number found after 10,000 iterations. In this approximation of the confidence interval, for sequences with the copy number <10 before sampling, it was impossible to determine whether the sequence disappeared due to random sampling or due to bias. The values of **Bias** operator cannot be defined for these sequences and it could be assumed to be 1 (see (11)). For copy number >10, however, sequence-specific bias can be readily detected. We observed that the removal of Phred <13 reads yielded a multiset in which a large number of sequences deviated beyond the confidence interval (Figure 6(d)). Their sequences could be readily extracted by comparing the vector ^13^ *n* with the vector of the lower confidence intervals ^lo^ *C* (see (12)). The solution of the **B** **i** **a** **s** can be illustrated graphically (Figure 6(e)). Top 30 censored sequences are listed in Table S1; the other sequences can be found in the supplementary information (file PhD7-Amp-0F-13F-CEN.txt).

We performed similar calculations for ^1^ *n* → ^30^ *n* and ^13^ *n* → ^30^ *n* processes. The latter process is the most interesting because ^13^ *n* library has all nucleotides within acceptable confidence range (>95%) and the distribution of cumulative quality suggested that errors, on average, do not cluster in one read (Figure 5). The ^13^ *n* → ^30^ *n* conversion eliminated 30% of the reads, and copy numbers of many sequences deviated significantly from the random sampling; these sequences are represented by green dots outside the blue confidence interval in Figure 6(h). Top 30 sequences are listed in Table S2. The censorship is not only sequence-specific, but also position-specific. In sequences that had been censored during the ^13^ *n* → ^30^ *n* process, lower quality reads clustered around 3-4 specific nucleotides (supplementary information Figure S5).

The mechanism that leads to the disappearance of censored sequences is not currently clear. We attempted to identify common motifs in censored sequences using two approaches: (i) clustering and principal component analyses based on Jukes-Cantor distance between sequences and (ii) identification of motifs using multiple unique sequence identifier software (MUSI) [17]. These approaches could not detect any property common to censored reads, which would make them significantly different from the other, noncensored reads. Still, we hypothesize that the observed censorship represents sequence-specific errors, which occur in every time such sequence passes though the Illumina analyzer. For example, the sequences listed in Tables S1 and S2 and supplementary files were censored in five independent experiments, which were pooled and processed simultaneously in one Illumina run. Analysis of other instances of Illumina sequencing performed by other groups could help prove (or disprove) that censorship is indeed sequence-specific and experiment-independent. Sequence-specific censorship during Illumina analysis has been described in other publications [46]. The observations presented above suggest that reading of some sequences in phage libraries does not yield an accurate copy number. Even if these sequences were enriched due to binding, their apparent copy number in sequencing would be decreased due to sequencing bias. If the magnitude of bias is known, however, such error could be corrected. We anticipate that other biases could be calculated for these and other libraries in similar fashion. Their calculation extends beyond the scope of this paper and it will be performed in our next publication.

## 3. Discussion

### 3.1. Significance and Transformative Potential of Library-Wide Error Correction

In the Medicinal Chemistry field, structure-activity relationships (SAR) and pharmacophores are built using both positive and negative observations. It is the negative results that bear the most significance in these studies because they allow mapping of the range of conditions under which particular structure no longer works. For example, SAR of an R group of a ligand might be built on the following observations. A ligand binds to the target when the R group in the specific position is methyl or ethyl; changing R to *iso*-propyl and *tert*-butyl ablates the binding. This concludes that the R group must be a small alkyl group. An analogous situation is found in SAR of peptide ligands; the most important information from alanine scan mutagenesis is loss of function because it helps identifying the important residues. Interestingly, loss-of-binding conclusions are never applied to phage-display. The phage-display field is driven by positive results. Most publications report and follow up only on sequences enriched in the screen and consider only large copy numbers interesting. All papers focus on sequences that were found. Very few papers in phage display ask why other sequences were not found.

One of the reasons why phage display is not used for SAR-type analysis is because negative observations in phage library cannot be determined with high confidence. From a practical point of view, measuring zero with high confidence requires the largest number of observation (the highest depth of sequencing). The payoff, however, is immense: one screen with “confident zeros” could potentially yield SAR for every possible substitution of every possible amino acid. We refer to this (theoretical) possibility as “Instant SAR,” and its condensed theoretical form is described in (3) or (9) and (10). This paper demonstrates that the depth of sequencing is not the only problem towards this goal. Accurate estimate of negative results requires complete characterization of the origins of errors in sequencing which yield false negative values by censoring certain sequencing. Other types of censorship, such as growth bias, should be characterized and eliminated as well. As the phage display field is currently focused on positive results, the need for optimal error corrections and recovery of erroneous reads is low. With the rise of SAR-type applications in phage display, error correction will be recognized as the most significant barrier because it could lead to improper assignment of low frequencies and negative results. Improved error correction strategies could assign a lower confidence to the sequence instead of eliminating the errors and labeling them as confident zero. Proper mathematical framework, possibly similar to the one used in this paper, could be then used to carry all confidence intervals through calculations to yield reliable SAR-type data.

We note that the framework described in this paper is suitable for the analysis of the selection from libraries in which the diversity of the libraries before and after selection could be covered entirely by deep sequencing. With the current depth of sequencing, it corresponds to medium-scale libraries of ~10^6^ random members and affinity-matured libraries that contain ~10^6^ point mutations. We are in the process of generating these medium-scale libraries and running selection procedures that will allow us to apply and refine our framework. In the future, as technical capabilities and depth of sequencing increase, the process would be applicable to larger libraries as well.

## 4. Methods

### 4.1. Generation of Z-Bars and Other Visualization Techniques

Sequencing of the libraries has been described in our previous publications [6, 47]. All data visualization in this paper was done by MATLAB scripts; raw *.eps output from MATLAB scripts subject to minor postprocessing in Adobe Illustrator to adjust fonts relative dimensions of plots. Core scripts are described in the supplementary information. Other scripts are available in our previous publication [47]. Illumina files used for the analysis can be found in the directory at http://www.chem.ualberta.ca/~derda/mathbiology/; the file ERROR_TAG_data0001.txt is an example of error-tagged reads; PhD7-Amp-xxF.txt is the library filtered with xx Phred cutoff (xx = 1, 13 and 30); file PhD7-Amp-13F-30F-CO.txt describes confidence intervals for Phred(13) to Phred(30) filtering process; other files with *-CO.txt extension describe confidence intervals of other processes. Supplementary Figures S1–S4 and Schemes S1 and S2 describe MATLAB implementation of the **S** **a** operator.

## Supplementary Material

Figure S1. Testing the performance of random indexing function.
Scheme S1. Matlab scripts of random indexing functions and scripts used to generate figure S1.
Scheme S2. Implementation of sampling operator acting on Fre vector.
Scheme S3. Implementation of the operator that calculates confidence interval; scripts has rudimentary error check features and progress display (waitbar).
Figure S2. Estimation of the total solutions for the Sa(M) operator, where M is a multiset, using rate of convergence.
Figure S3. Properties of the Sa(M) solutions for multisets that have elements with very different copy numbers.
Figure S4. Visualization of the Sa(M) solutions for sparse multiset with [1 2 4 8 16 32 64] frequency vector on linear and log scale.
Figure S5. Distribution of errors in censored reads.
Table S1. Top 30 sequences censored during the ^13^n → ^30^n process.
Table S2. Top 30 sequences censored during the ^1^n → ^13^n process.

## Figures and Tables

**Table 1:** Symbols and definitions used in the theoretical description section.

Symbols	Meaning
A, a, f, m, n, k	Unless specified otherwise, normal font designates scalars
*A*, *a*, *N*, *P*,_ _^1^n, _ _^13^n	Italic font designates vectors. Different vectors can be distinguished by the left-superscript notation
**A, a, Abc, Pan, Sa**	Bold font designates operators or matrices (here all operators are matrices)
^1^ **A**,^**f**^ **S** **a**,^0.9^ **S** **a**,^0.5^ **S** **a**	Operators can be distinguished by the left-superscript notation. For sampling operator **Sa**, this notation specifies the sampling fraction of the **Sa** operator
A_1_, a_2_, A_*i*_, a_*j*_	Normal font with right subscript designates scalar values of the vector
A_11_, A_21_, A_ij_, A_ii_	Normal font with two right subscripts designates scalar values of the 2D matrix
||A_1_ … A_5_||	Description of the scalar elements in the vector
||A_ij_ … A_ii_||	Description of the scalar elements in the matrix
x *∈* [A B]	Scalar x belongs to the inclusive scalar interval [A B]; that is, A ≤ x ≤ B
*x* ∈ [*A* *B*]	Vector *x* belongs to the “vector interval” [*A* *B*]; that is, for every element A_i_ ≤ x_i_ ≤ B_i_
{A B C … X}	Set where A, B, C,…, X are the unique elements of the set
{A(a) B(b)…X(x)}	Multiset (2-tuple) where A, B,…, X are the unique elements and a, b, x are the scalars describing the copy numbers of the A, B, X elements
I_N_	Unity matrix of the Nth order; that is, N × N matrix ||A_ij_||, A_ij_ = *δ*_ij_ (Kronecker delta)
