# Supplementary material for: Error Analysis of Deep Sequencing of Phage Libraries: Peptides Censored in Sequencing
Source: Comput Math Methods Med. 2013 Dec 12;2013:491612. doi: 10.1155/2013/491612 (PMC3876701; doi:10.1155/2013/491612)
Supplement: Supplementary file 1 — Figure S1. Testing the performance of random indexing function. Scheme S1. Matlab scripts of random indexing functions and scripts used to generate figure S1. Scheme S2. Implementation of sampling operator acting on Fre vector. Scheme S3. Implementation of the operator that calculates confidence interval; scripts has rudimentary error check features and progress display (waitbar). Figure S2. Estimation of the total solutions for the Sa(M) operator, where M is a multiset, using rate of convergence. Figure S3. Properties of the Sa(M) solutions for multisets that have elements with very different copy numbers. Figure S4. Visualization of the Sa(M) solutions for sparse multiset with [1 2 4 8 16 32 64] frequency vector on linear and log scale. Figure S5. Distribution of errors in censored reads. Table S1. Top 30 sequences censored during the 13n → 30n process. Table S2. Top 30 sequences censored during the 1n → 13n process. [file 491612.f1.pdf]

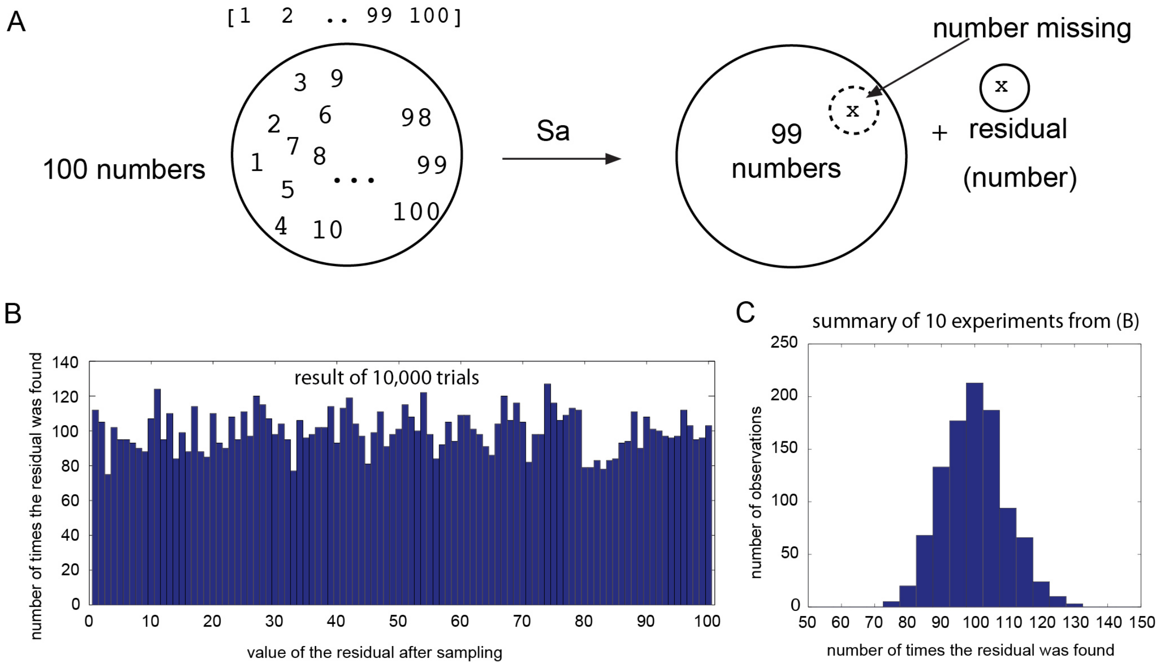

Figure S1. Testing the performance of random indexing function.

```
% This script tests the random index generator function by looking at
% the distribution of residuals in 10 sets of 10,000 trials
X=[]; Y=[]; Nout=[];

for kk=1:10
    for i=1:10000
        X(i)=i;
        [~,Y(i)]=randN(99,100);
    end
    figure(1); % pannel B
    [nout] = hist(Y,0:1:100);
    hist(Y,1:1:100);
    figure(2); % pannel C
    Nout=[Nout nout];
end

function [out,residual] = randN(n,N)
% Generate random n integers from the set of N integers; n<N

    t2=0; out=[]; N1=1:1:N;

    while t2<n
        % generate random integers
        t1 = floor(random('unif',1, numel(N1)+1-1e-5, 1, n-t2));
        tU = unique(t1);        % discard repeating elements
        out=[out N1(tU)];       % combine with previous elem.
        t2 = numel(out);        % count current elements
        N1=setdiff(N1,N1(tU)); % remove the found elements
    end
    residual=N1;
end
```

Scheme S1. Matlab scripts of random indexing functions and scripts used to generate figure S1.

```
function [SaFre] = SA(Fre,n)
    % Generates a random sample of the frequency vector.

    [EX,U]= expand(Fre);         % Create an expanded multiset.
    IX    = randN(n,sum(Fre));   % Generate random index vector
    sFre  = EX(IX);              % Create random sample
    SaFre = fold(U,Fre,sFre);    % fold back to frequency form

end

function [out,residual] = randN(n,N)
% Generate random n integers from the set of N integers; n<N

    t2=0; out=[]; N1=1:1:N;

    while t2<n
        % generate random indices
        t1 = floor(random('unif',1, numel(N1)+1-1e-5, 1, n-t2));

        tU = unique(t1);       % discard repeating elements
        out=[out N1(tU)];      % combine with previous elem.
        t2 = numel(out);       % count current elements
        N1=setdiff(N1,N1(tU)); % remove the found elements
    end
    residual=N1;
end

function [EXP, U] = expand(Fre)
% Expand multiset from a frequency form
    EXP = zeros(1, sum(Fre));  % blank expanded multiset
    U = 1:1:numel(Fre);        % unique identifiers

    h = waitbar(0,'');
    binE=0;
    for i=1:numel(Fre)
        binE = binE+Fre(i);     % bin end
        binS = binE-Fre(i)+1;   % bin start
        EXP(binS:binE)=U(i); % fill each bin
        if mod( i, round(numel(Fre)/100) )==0
            waitbar(i/numel(Fre),h,'converting multiset');
        end
    end
    delete(h);
end

function [SaFre] = fold(U,Fre,sFre)
% collapse the expanded multised to frequency form.
    temp1 = sort(sFre, 'ascend')';
    [found, temp2, ~] = unique(temp1, 'first');

    temp3     = [temp2(2:end); numel(temp1)+1];
    Occur = temp3-temp2;
    clear temp*

    [~,notfound]    = setdiff(U,found);
    SaFre           = Fre;
    SaFre(notfound) = 0;
    SaFre(found)    = Occur;
end
```

Scheme S2. Implementation of sampling operator acting on Fre vector.

```
function [C] = confidint(Fre,n,nn)
    % Generates a confidence interval of the sampling operator
    % Frequency vector must be Nx1 if not, transpose it
    if size(Fre,1)~=1
        if size(Fre,2)==1
            Fre=Fre';
            TRANSPOSE=1;
        else
            error('vector must be Nx1 or 1xN !! ');
        end
    else
        TRANSPOSE=0;
    end

    [EX,U]= expand(Fre);        % Create an expanded multiset.

    Ave = round(Fre*(n/sum(Fre)));
    C   = [Ave; Ave];           % seed using average values
    MAX = find(Fre==max(Fre));
    M   = num2str(Ave(MAX));

    % repeat sampling nn times
    hh = waitbar(0,'1','Name',['Calculating confidence interval'...
                'in ' num2str(nn) ' steps'],...
                'CreateCancelBtn','setappdata(gcbf,''canceling'',1)');

    for kk=1:nn
        if getappdata(hh,'canceling')
            break
        end

        IX    = randN(n,sum(Fre));  % Random index vector
        sFre  = EX(IX);             % Create random sample
        SaFre = fold(U,Fre,sFre);   % Fold to frequency form

        C (1, SaFre<C(1,:) ) = SaFre ( SaFre<C(1,:) );
        C (2, SaFre>C(2,:) ) = SaFre ( SaFre>C(2,:) );

        if mod(kk,round(nn/100))==0
            str = ['Interval for maximum value is [ '...
                   num2str(C(1,MAX)) ' ...' M '...  ' ...
                   num2str(C(2,MAX)) ' ]'];
            waitbar(kk/nn,hh,sprintf('%c',str));
        end
    end
    delete(hh);

    if TRANSPOSE C=C'; end
end
```

Scheme S3. Implementation of the operator that calculates confidence interval; scripts has rudimentary error check features and progress display (waitbar). Note that this implementation required `randN`, `expand` and `fold` functions (see Scheme S2 for code)

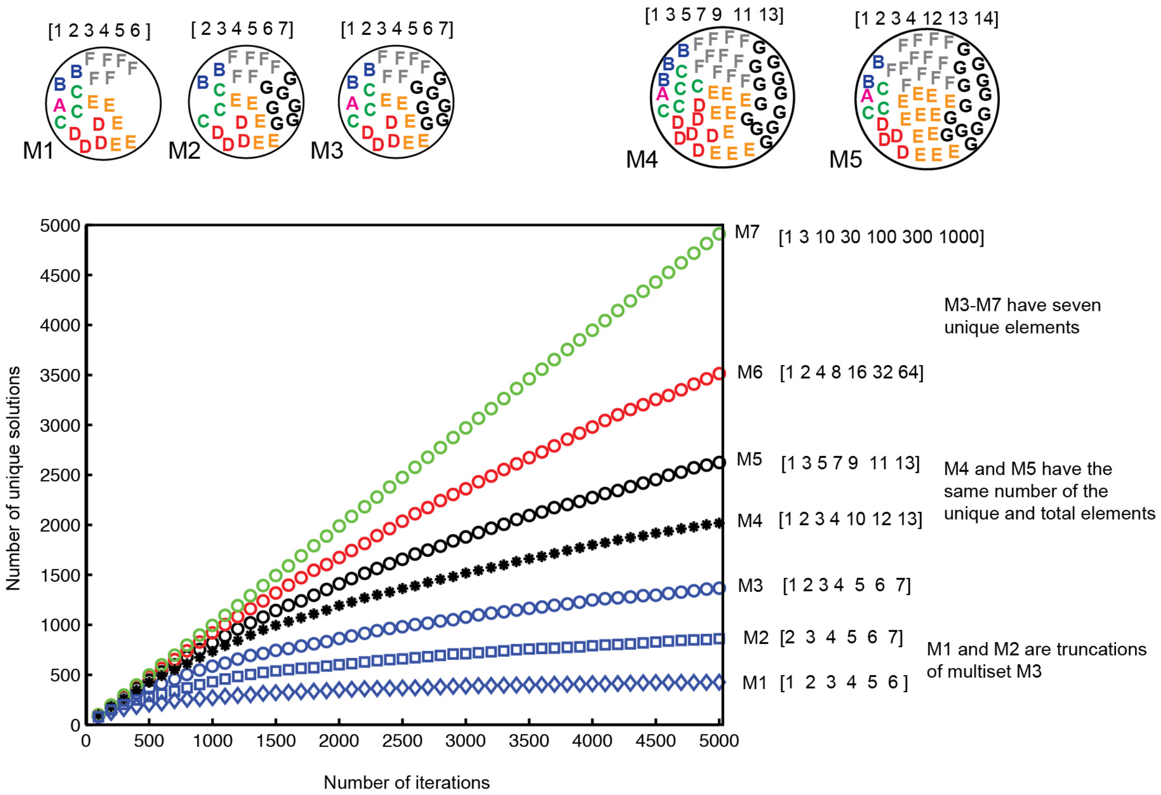

Figure S2. Estimation of the total solutions for the **Sa**(M) operator, where M is a multiset, using rate of convergence. We examined several multisets that contain six or seven unique elements with different copy numbers (some are represented graphically). Number of unique solutions for the **Sa** operator depends on the unique and total size, as well at the internal structure of the multiset. For example, M4 and M5 have identical number of unique and total elements but different estimated number of **Sa**(M4) and **Sa**(M5).

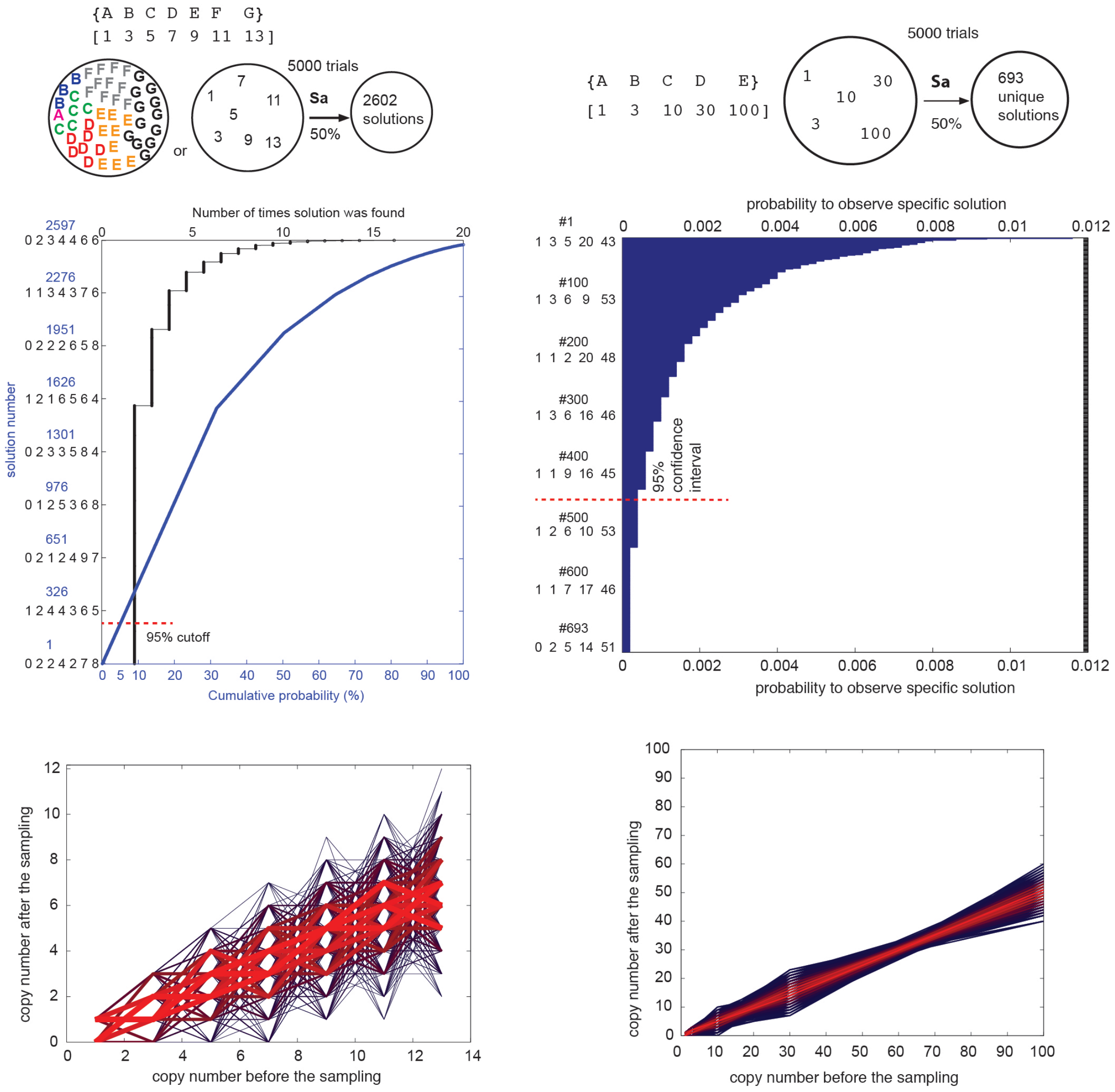

Figure S3. Properties of the **Sa**(M) solutions for multisets that have elements with very different copy numbers. Collections of probable solutions has biconcave or "pine cone" shape indicating that no solution contains more than one outlier point. While this property is true to sampling of any multiset, this property is easier to visualize in these "sparse" multisets.

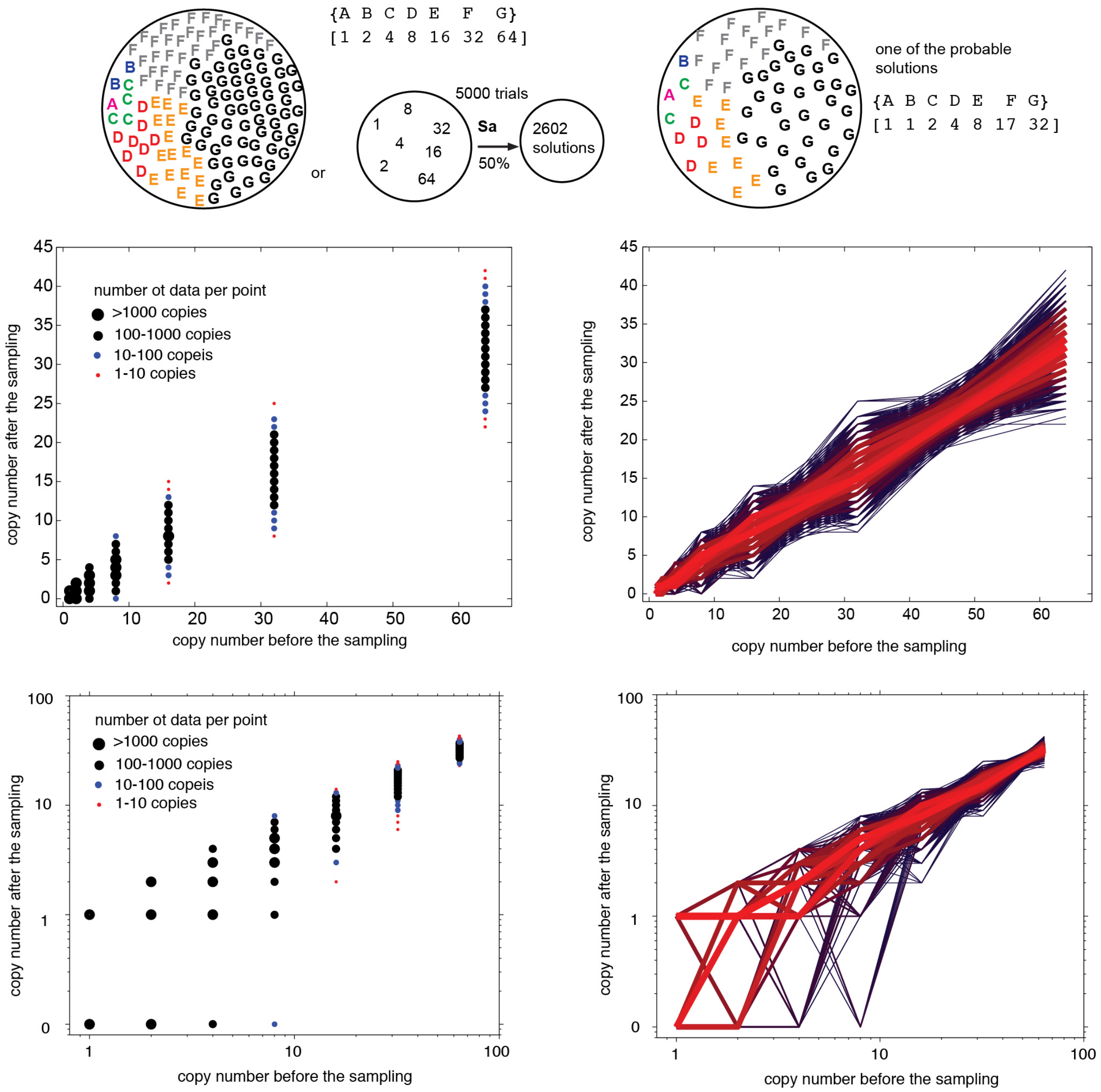

Figure S4. Visualization of the **Sa**(M) solutions for sparse multiset with [1 2 4 8 16 32 64] frequency vector on linear and log scale. Lines of different thickness (right) denote solutions found with different probability to find a specific vector, while dots of different size (left) denote probabilities of finding the individual frequencies in the vector. The “outlier frequencies” represented by blue dots describe >99% confidence boundary of each frequency. These solutions have high confidence because they were found >10 times in 5000 iterations. No solution in 5000 iterations connects two “outlier” frequencies. Blue outliers, thus, describe a region in which can be described as “1% of the solutions contains only <u>one</u> of the blue outliers”. Probability of finding the solution that contains exactly <u>one</u> red outlier is between 0.02% and 0.2%. The other frequencies in these improbable solutions reside in the “probable interval” (denoted by red lines).

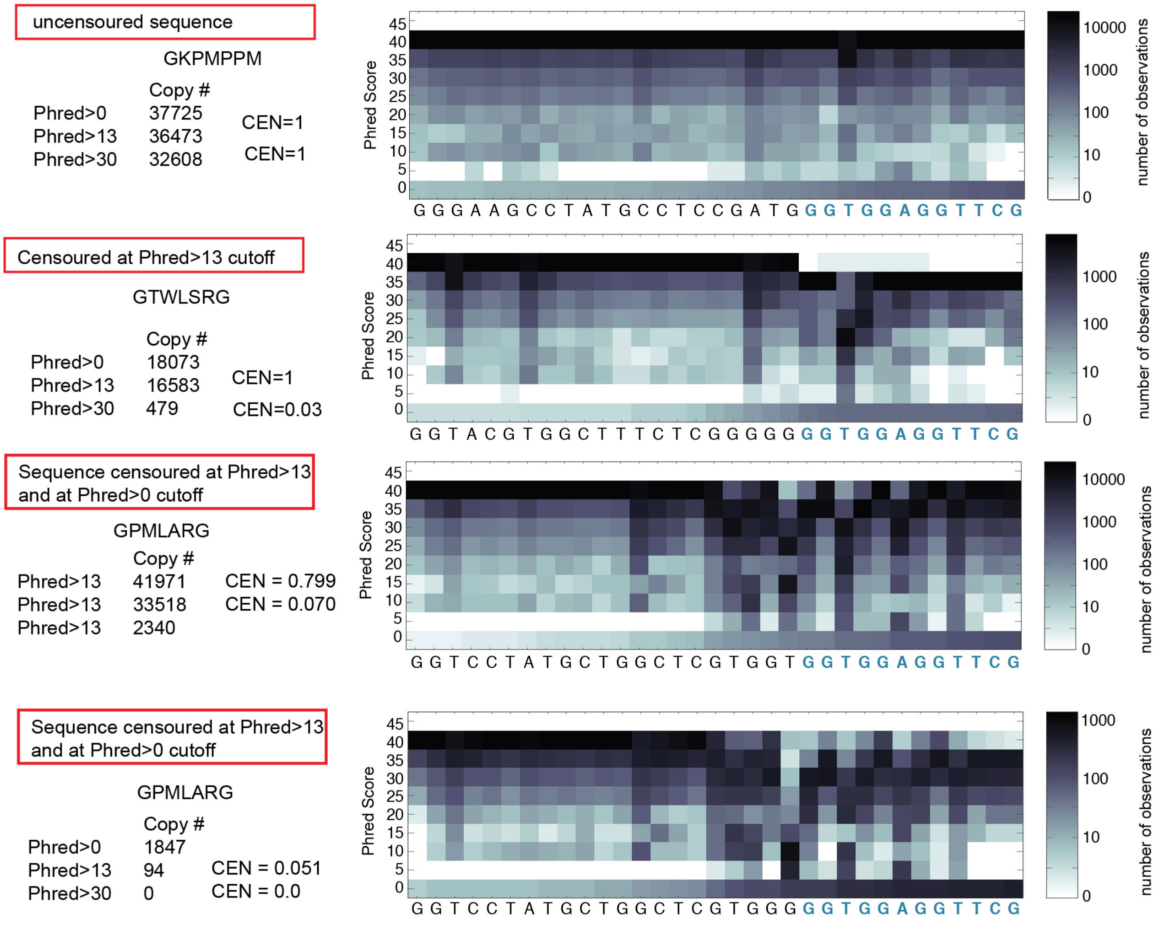

Figure S5. Distribution of errors in censored reads. In uncensored sequences (top) the errors occur at low abundance uniformly throughout the whole read. In censored reads, errors occur preferentially in 3-4 specific nucleotides.

Nucleotide	peptide	^{13}n	^{30}n	^{1o}C	CEN
GGTCCTATGCTGGCTCGTGGT	**GPMLARG**	33518	2340	24732	0.070
GGTAAGGTGCAGGCGCAGTCG	GKVQAQS	24566	13168	18101	0.536
CAGCTGATGAATGCTTCGCGG	QLMNASR	21821	10957	16070	0.502
ATGCTGCCGTCTGTGCTTGAT	MLPSVLD	17619	12446	12955	0.706
GGTACGTGGCTTTCTCGGGGG	GTWLSRG	16583	479	12154	0.029
CAGAGTCCTGATGAGGTTTGG	QSPDEVW	14482	8780	10615	0.606
GCGACGCCGTCGTGGTGGGCT	ATPSWWA	13658	8031	9997	0.588
ACGACGCGTCTTCCGGTTATT	TTRLPVI	11538	8063	8446	0.699
GCGCGTCCGCCTCTGTTTGGT	ARPPLFG	11436	7745	8371	0.677
TGGCCTACGCTGCAGTGGGCG	WPTLQWA	11097	5017	8112	0.452
AGTCAGACGAAGGTGCCGTTG	SQTKVPL	10129	6138	7380	0.606
ACGCTGTTGCAGGCGGCTAGG	**TLLQAAR**	9819	2841	7144	0.289
AATCAGCAGCCGGCTCCTCGG	NQQPAPR	7634	5187	5566	0.679
CGGCTTCCGTCTTGGCATGAG	**RLPSWHE**	7587	3867	5533	0.510
GCTGCTAAGACGCCTACGGAG	**AAKTPTE**	7468	2987	5442	0.400
CTACCTTCATATCATGTGCCT	**LPSYHVP**	7410	4557	5394	0.615
GATGCGGGGTATGTGACTTTG	**DAGYVTL**	7410	4103	5394	0.554
GCGACGACTGTTCCAGCTTCG	ATTVPAS	7287	4514	5290	0.619
AAGCTTCCTGGGTGGTCGGGG	KLPGWSG	6832	340	4970	0.050
GCGTCTACGTTGAAGTGGGCG	ASTLKWA	6776	2279	4928	0.336
AAGCCGGTTCAGCTGGATCAT	KPVQLDH	6744	4687	4906	0.695
GGGGAGACTCGTGCGCCGCTT	**GETRAPL**	6680	4803	4850	0.719
AATCCGATGCAGTCTCGTCCG	NPMQSRP	5928	4135	4297	0.698
TCGTATGCGTCGGAGAAGCGT	SYASEKR	5804	3838	4221	0.661
ACGCCGCAGTGGGCTGGTCAG	**TPQWAGQ**	5602	3638	4063	0.649
ACGCGGGCTGGTCTGGATTTT	TRAGLDF	5538	3275	4007	0.591
CAGCGGCTGCCTCAGACGGCG	**QRLPQTA**	5483	2	3973	0.000
TGGACTGGTTCGTATAGGTGG	**WTGSYRW**	5174	2239	3738	0.433
CATCATGCGCTGCGTTTGGAG	HHALRLE	4993	3196	3610	0.640

Table S1. Top 30 sequences censored during the $^{13}n \rightarrow {}^{30}n$ process. Bolded sequences could also be found in censorship during the $^{1}n \rightarrow {}^{13}n$ process (partially described in Table 2). Normal-font sequences are uniquely censored in $^{13}n \rightarrow {}^{30}n$ process. While typical censorship is a factor of two or three, the highlighted reads are censored by a factor of 10 or more.

Nucleotide	peptide	^{13}n	^{30}n	^{10}C	CEN
GGTCCTATGCTGGCTCGTGGT	**GPMLARG**	41971	33518	38273	0.799
CATGTGCTTCGTTTTGATACG	HVLRFDT	30513	27073	27804	0.887
CATGTGAAGCCTCTGGTGACG	HVKPLVT	18102	16266	16451	0.899
ACGCTGTTGCAGGCGGCTAGG	**TLLQAAR**	11108	9819	10095	0.884
CAGCGGCTGCCTCAGACGGCG	**QRLPQTA**	10687	5483	9667	0.513
CGGCTTCCGTCTTGGCATGAG	**RLPSWHE**	8794	7587	7966	0.863
GCTGCTAAGACGCCTACGGAG	**AAKTPTE**	8445	7468	7628	0.884
CTACCTTCATATCATGTGCCT	**LPSYHVP**	8442	7410	7640	0.878
GATGCGGGGTATGTGACTTTG	**DAGYVTL**	8241	7410	7452	0.899
GGGGAGACTCGTGCGCCGCTT	**GETRAPL**	7546	6680	6834	0.885
CATGGGCTGTCTCATCGGCTT	**HGLSHRL**	6793	4034	6136	0.594
ACGAGTCCTCGGATTGCGCCT	TSPRIAP	6370	5721	5761	0.898
ACGCCGCAGTGGGCTGGTCAG	**TPQWAGQ**	6244	5602	5641	0.897
TGGACTGGTTCGTATAGGTGG	**WTGSYRW**	5839	5174	5267	0.886
AGTCTGAGGCATGGGTCGTAT	**SLRHGSY**	5401	4425	4882	0.819
TCGGTGGAGTCGGCGTGGAGG	**SVESAWR**	5104	4408	4604	0.864
TCGCCTCATTTGCATGGGGCT	SPHLHGA	4674	4170	4219	0.892
CTGGCGCGTGAGCCTACGTCG	**LAREPTS**	4215	3747	3800	0.889
CATACGGTTCGGACTGGTGAG	**HTVRTGE**	4154	3617	3738	0.871
TCGCGGACTTTGATTGCGCCG	**SRTLIAP**	3620	3236	3258	0.894
GCGGCTGGTCAGCAGTTTCCT	**AAGQQFP**	3510	2790	3151	0.795
GCGACGGGTTGGTCTGCGTTG	**ATGWSAL**	3477	3087	3131	0.888
TCGGAGGCTGAGGCGACGTAT	**SEAEATY**	3389	3023	3039	0.892
CATGTGTATGAGTTTGGGCCG	**HVYEFGP**	3311	2877	2977	0.869
CTTGTGACGACGTGGCCGGCT	**LVTTWPA**	3116	2721	2787	0.873
ACGGGTGTGACGCTTACGGTG	**TGVTLTV**	3111	2437	2791	0.783
GAGTATCGGCTGCTTTATTCG	**EYRLLYS**	2968	1955	2666	0.659
GCGGCGTGGCAGCTTCATAGT	**AAWQLHS**	2801	2491	2515	0.889
TCGGCTACTCAGGCTTCTGTG	**SATQASV**	2791	2356	2501	0.844
CAGGAGCCGCTTCCTGCTTTG	QEPLPAL	2492	2166	2237	0.869
ACGGCGCGGTATCCGTCGTGG	**TARYPSW**	2199	1959	1962	0.891
AATACTGATGTTGCTGGTGGT	**NTDVAGG**	2180	1919	1944	0.880
CAGGCGGGGCTTCTGCGTCAT	**QAGLLRH**	2149	1876	1922	0.873
CGGGCTGATATGTCGACTGTG	**RADMSTV**	2098	1858	1878	0.886
TGGGGGGGGCTGCCTGAGCCT	**WGGLPEP**	2047	1591	1817	0.777
GGTCCTATGCTGGCTCGTGGG	**GPMLARG**	1847	94	1646	0.051

Table S2. Top 30 sequences censored during the $^{1}n \rightarrow {}^{13}n$ process. Bolded sequences can also be found in censorship during the $^{13}n \rightarrow {}^{30}n$ process (partially described in Table 1). Red sequences are uniquely censored in $^{1}n \rightarrow {}^{13}n$ process.
